# Influence of 16S rRNA Hypervariable Region on Estimates of Bacterial Diversity and Community Composition in Seawater and Marine Sediment

**DOI:** 10.3389/fmicb.2019.01640

**Published:** 2019-07-16

**Authors:** Zak Kerrigan, John B. Kirkpatrick, Steven D’Hondt

**Affiliations:** ^1^Graduate School of Oceanography, The University of Rhode Island, Narragansett, RI, United States; ^2^The Evergreen State College, Olympia, WA, United States

**Keywords:** deep life, deep biosphere, marine sediment, marine water column, tag sequencing, bacteria, C-DEBI

## Abstract

To assess the influence of 16S ribosomal RNA (rRNA) tag choice on estimates of microbial diversity and/or community composition in seawater and marine sediment, we examined bacterial diversity and community composition from a site in the Central North Atlantic and a site in the Equatorial Pacific. For each site, we analyzed samples from four zones in the water column, a seafloor sediment sample, and two subseafloor sediment horizons (with stratigraphic ages of 1.5 and 5.5 million years old). We amplified both the V4 and V6 hypervariable regions of the 16S rRNA gene and clustered the sequences into operational taxonomic units (OTUs) of 97% similarity to analyze for diversity and community composition. OTU richness is much higher with the V6 tag than with the V4 tag, and subsequently OTU-level community composition is quite different between the two tags. Vertical patterns of relative diversity are broadly the same for both tags, with maximum taxonomic richness in seafloor sediment and lowest richness in subseafloor sediment at both geographic locations. Genetic dissimilarity between sample locations is also broadly the same for both tags. Community composition is very similar for both tags at the class level, but very different at the level of 97% similar OTUs. Class-level diversity and community composition of water-column samples are very similar at each water depth between the Atlantic and Pacific. However, sediment communities differ greatly from the Atlantic site to the Pacific site. Finally, for relative patterns of diversity and class-level community composition, deep sequencing and shallow sequencing provide similar results.

## Introduction

Over the last 10 years, there has been a large increase in literature on microbial community composition in marine sediment (Inagaki et al., 2006, 2015; Blazejak and Schippers, 2010; Biddle et al., 2012; Briggs et al., 2012; Durbin and Teske, 2012; Mills et al., 2012; Breuker et al., 2013; Colwell and D’Hondt, 2013; Orsi et al., 2013, 2017; Lloyd, 2014; Teske et al., 2014; Bienhold et al., 2016; Brandt and House, 2016; Jones et al., 2016; Nunoura et al., 2016; Labonté et al., 2017; Petro et al., 2017; Harrison et al., 2018; Orsi, 2018), and how it compares to community composition in the overlying water (Quaiser et al., 2011; Hamdan et al., 2013; Walsh et al., 2016b). Seawater and marine sediment contain roughly equal numbers of microbial cells (Kallmeyer et al., 2012), but bacterial taxonomic richness is generally much higher in the ocean than in the sediment (Walsh et al., 2016b). The ability to characterize these communities in detail is due to the advent of high-throughput sequencing (HTS) technology, which allows sequencing of either a large number of samples on a single sequencing run, or deep sequencing of a small number of samples (hundreds of thousands to millions of amplicons per sample). Consequently, HTS is now the dominant method for analyzing environmental microbiology because it is able to capture a large portion of the community in each sample. However, HTS is limited by its capacity to sequence relatively short regions of DNA, and therefore relies on analysis of only one or two of the hypervariable regions of the 16S ribosomal RNA (rRNA) gene to identify taxonomic diversity and community composition. Most recent studies of microbial diversity in seawater and marine sediment typically analyzed a single 16S hypervariable region (e.g., Inagaki et al., 2006; Sogin et al., 2006; Huber et al., 2007, Mills et al., 2012; Gibbons et al., 2013; Bienhold et al., 2016; Brandt and House, 2016; Jones et al., 2016; Nunoura et al., 2016; Walsh et al., 2016b; Labonté et al., 2017; Orsi et al., 2017; Starnawski et al., 2017; Harrison et al., 2018).

While HTS has become a valuable tool in microbial ecology, previous studies have shown that estimates of taxonomic diversity and community composition depend on which hypervariable region is analyzed (Liu et al., 2008; Youssef et al., 2009; Mysara et al., 2017). This dependence is problematic because different studies have analyzed different regions [V3 from Mills et al. (2012); V4 from Starnawski et al. (2017) and Orsi et al. (2017); V6 from Huber et al. (2007) and Bienhold et al. (2016); V1–V3 from Harrison et al. (2018); V2–V5 from Inagaki et al. (2006); V4–V6 from Walsh et al. (2016b); V5–V6 from Jones et al. (2016); and V6–V9 from Brandt and House (2016)]. Reliance of different studies on different regions hinders the synthesis of these studies to draw broad conclusions about distributions of microbial richness and community composition in the open ocean and marine sediment.

To determine what information from a 16S study is consistent across hypervariable regions and help provide a stable foundation for synthesizing results from studies of different hypervariable regions, we separately amplified both the V4 and V6 hypervariable region of the bacterial 16S rRNA gene from multiple horizons in the ocean and marine sediment of the Atlantic and Pacific Oceans. Sampled horizons range from the surface, sunlit water to 5.5-Ma subseafloor sediment in individual sites from both oceans. Our goals are to (i) assess the extent to which interpretations of marine microbial diversity and community composition depend on the hypervariable region chosen, and (ii) identify the results that can be used to make conclusions about these communities regardless of hypervariable region.

## Materials and Methods

### Sample Collection and DNA Extraction

We collected samples from water-column filters and sediment cores from Site 8 of R/V Knorr cruise 195-3 (00°00.36′N 147°47.50′W, water depth 4360 m) in the Equatorial Pacific and Site 15 of R/V Knorr cruise 223 (33°29.01′N 054°09.98′W, water depth 5510 m) in the North Atlantic. At each site, we collected seven samples; four water-column samples at depths corresponding to the Chlorophyll-a maximum (*Chl-a*), oxygen minimum zone (OMZ), bulk deep water, and bottom water, and three sediment samples at depths from the sediment–water interface, sediment dated approximately 1.5 million years old (Ma), and sediment dated approximately 5.5 Ma. We extracted DNA from both the water filters and sediment with the MOBIO PowerSoil DNA Isolation Kit (Mo Bio Laboratories, A Qiagen Company, Carlsbad, CA, United States), following the manufacturer’s protocol. For the water-column samples, we filtered 10 L of seawater through 0.22 μm Sterivex filters (Millipore Sigma, Billerica, MA, United States), then shredded each filter and placed the pieces in two of the MOBIO PowerSoil bead tubes for extraction using the standard PowerSoil protocol. For all but two sediment samples, we used duplicate subsamples of 0.25 g of sediment for DNA extraction. For the remaining two sediment samples [taken from 7 m below sea floor (mbsf) (1.5 Ma) and 26 mbsf (5.5 Ma) at Site 8], we used 12 subsamples of 0.25 g of sediment due to the extremely low biomass of the material. In addition to the sediment samples, the entire PowerSoil extraction protocol was completed on an empty sample tube to analyze for lab and kit contamination during post-sequencing processing. Sediment age was approximated for each depth by dividing basement age (Müller et al., 2008) by sediment thickness (Divins, 2003), and assuming a constant sediment deposition rate.

### PCR Amplicon Construction and Sequencing

From each extract, we amplified the V4 and V6 hypervariable regions of the 16S rRNA gene using forward and reverse primers from Caporaso et al., 2011, and the Visualization and Analysis of Microbial Population Structures (VAMPS) center^1^, respectively. The V4 primers are 515F (5′-GTGCCAGCMGCCGCGGTAA-3′) and 806R (5′-GGACTACHVGGGTWTCTAAT-3′). The V6 primers are a combination of four forward primers, 967F1 (5′-CTAACCGANGAACCTYACC-3′), 967F2 (5′-CNAC GCGAAGAACCTTANC-3′), 967F3 (5′-CAACGCGMARAAC CTTACC-3′), and 967F4 (5′-ATACGCGARGAACCTTACC-3′), and one reverse primer, 1064R (5′-CGACRRCCATGC ANCACCT-3′). We performed a 20-μl PCR reaction in triplicate for each sample, in which each reaction contained a mixture of 0.1 μl Platinum HF Taq Polymerase (Life Technologies, Carlsbad, CA, United States), 2 μl Platinum HF Buffer (10×), 0.8 μl MgSO_4_ (50 mM), 0.16 μl dNTPs (25 Mm mix), 0.1 μl of each primer (50 μM), 0.1 μl bovine serum albumin (Fermentas Life Sciences, Carlsbad, CA, United States), and between 1 and 10 μl of the extracted DNA in addition to a reaction containing 10 μl of water, and no extract to analyze for PCR reagent contamination during post-sequencing processing. The thermocycler program for both the V4 and V6 regions was set to an initial denaturation temperature of 94°C for 2 min; 30 cycles (37 cycles for sediment depths 7 and 26 mbsf at Site 8) of 94°C for 15 s, 60°C for 30 s, and 68°C for 60 s; and then a final extension of 68°C for 5 min. We then pooled and cleaned the amplicons for each sample using the Agencourt AMPure PCR Purification kit (Beckman Coulter Life Sciences, Indianapolis, IN, United States), and then sequenced the amplicons on the Illumina MiSeq at the University of Rhode Island Genomics and Sequencing Center. Sequencing of the V4 and V6 datasets was conducted using Illumina V2 chemistry with 2 × 250 cycles and V3 chemistry with 2 × 75 cycles, respectively.

### Sequence Analysis

To process and analyze the sequences, we used mothur v.1.38.1 (Schloss et al., 2009), and followed the mothur MiSeq SOP (Kozich et al., 2013) (December 2016). In addition to the steps in the SOP, we removed from all samples any reads that were sequenced from the PowerSoil extraction kit blank and PCR blank (described above) to account for possible lab and reagent contamination. Prior to clustering, we randomly subsampled all samples to 230,097 sequences (lowest number of reads in any sample) to make direct comparisons when conducting community analyses. Unless otherwise stated, we performed an average-neighbor clustering analysis at 97% sequence similarity for both the V4 and V6 hypervariable regions. Additionally, we removed singletons and doubletons of operational taxonomic units (OTUs) from each sample to mitigate the likelihood of including OTUs based solely on sequencing error (NCBI BioProject PRJNA423041, Accession Numbers SAMN08204781–SAMN08204808).

## Results

### Taxonomic Richness

**FIGURE 1:**
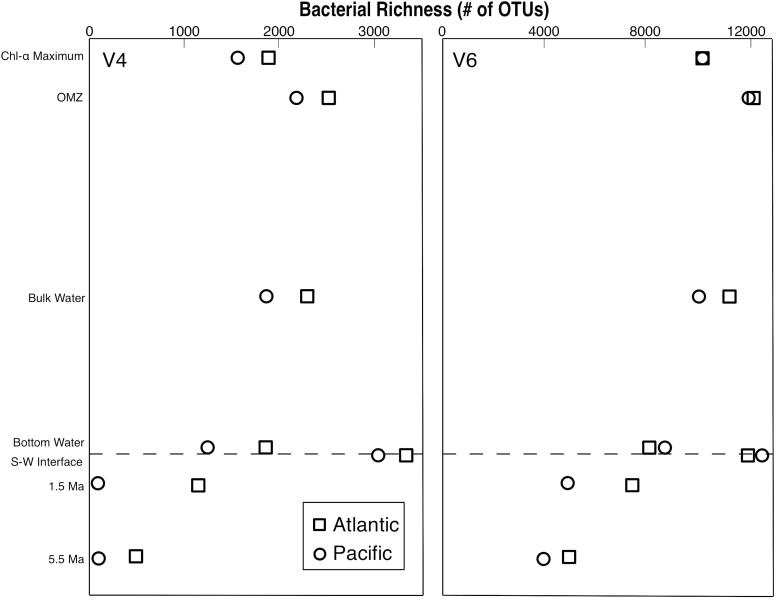
Bacterial richness profiles from the Pacific and Atlantic analyzed with both the V4 and V6 hypervariable region of 16S rRNA.

**FIGURE 2:**
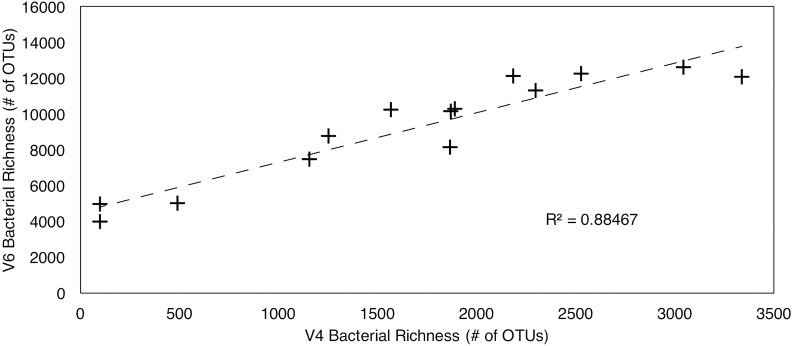
Linear regression of V4–V6 hypervariable region richness values. *R*^2^ = 0.88467.

In total, 12,316 OTUs were identified from all samples by clustering the V4 tags. 58,087 OTUs were identified by clustering the V6 tags. Despite the large difference in total number of OTUs from the two tags, the V4 and V6 tag profiles exhibit similar relative vertical patterns of taxonomic richness at both the Atlantic and Pacific sites (Figure 1). In each case, bacterial richness is highest in seafloor sediment 0–1 cm below the sediment–water interface, and then diminishes with increasing sediment age, consistent with established trends in the deep subseafloor (Walsh et al., 2016a,b). In the water column, richness is highest in the OMZ. These vertical distributions of relative richness in the water column broadly match distributions previously reported for the Southern Ocean and other Pacific sites, based on analysis of the entire V5–V8 and V4–V6 hypervariable region (Signori et al., 2014; Walsh et al., 2016b, respectively). To further illustrate the similarity in patterns of relative abundance, we directly compared the absolute numbers of bacterial richness between the V4 and V6 tags (Figure 2), and found a very strong correlation (*R*^2^ = 0.885).

### Taxonomic Evenness

**FIGURE 3:**
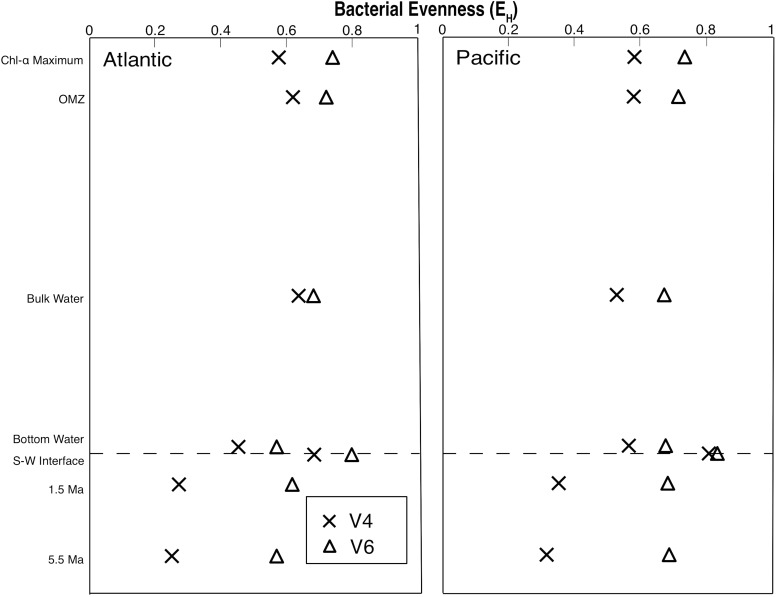
Bacterial evenness (E_H_) profiles from the Pacific and Atlantic analyzed with both the V4 and V6 hypervariable region of 16S rRNA.

For each sample, we calculated the Shannon’s Equitability Index (*E*_H_ = *H*/*lnS*), where *H* is the Shannon index and *S* is the taxonomic richness (total number of OTUs) for each sample. This results in a measure of OTU evenness on a scale from 0 to 1, where 1 is a value of total evenness (each OTU in the sample contains the same number of sequences). Although evenness is consistently higher with the V6 tag than with the V4 tag, both *E*_H_ profiles exhibit broadly similar vertical patterns, with highest evenness at the sediment–water interface (Figure 3). For all of the water column samples and the two seafloor sediment samples, the V6 *E*_H_ values are between 7 and 27% larger than the V4 values, indicating a more even distribution of OTUs. However, in the remaining subseafloor sediment of both the Pacific and Atlantic, the V6 *E*_H_ values are between 94 and 127% larger than the V4 values. This indicates that the subseafloor sediment communities are dominated by one or two V4-defined OTUs, but contain relatively even distributions of V6-defined OTUs.

### Community Composition

#### Similarity/Dissimilarity of Community Compositions From Different Samples

**FIGURE 4:**
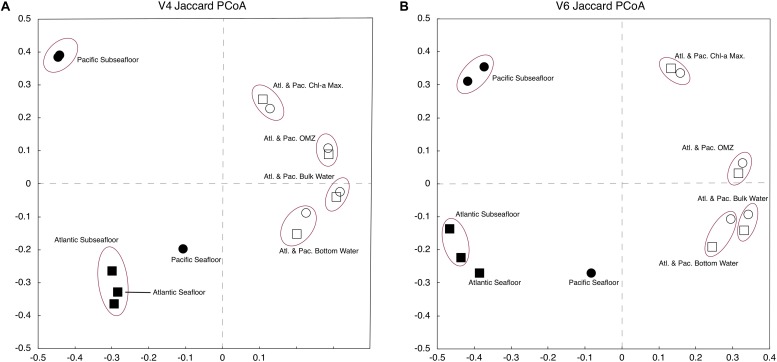
Jaccard principal coordinate analysis (PCoA) of V4-based **(A)** and V6-based **(B)** tag sequences. Atlantic samples are squares and Pacific samples are circles. The sediment is depicted with darkened markers, and the water column is depicted with open markers.

To compare the genetic similarity/dissimilarity between different samples, we completed a two-dimensional, principal coordinate analysis (PCoA). We calculated both Jaccard and Bray–Curtis distance matrices for use in the PCoA for comparison. As shown by the Jaccard results in Figure 4, PCoA of the V4-based OTUs and PCoA of the V6-based OTUs yield very similar sample groupings. For the Jaccard-based PCoAs (Figure 4), the two axes plotted explain 73 and 85% of the total variation for the V4-based and V6-based OTUs, respectively. The Bray–Curtis analysis (not shown) groups the samples similarly to the Jaccard analysis with a total variation of 54 and 57% explained by the first two axes for the V4-based and V6-based OTUs, respectively.

For both the V4-based and V6-based analysis, the water-column samples are more similar at each water depth regardless of geographic location (Figure 4). The subseafloor samples resemble each other closely within each geographic location, but differ greatly from one location to the other (Figure 4). These results are consistent with other studies conducted in the water column using the V6 rRNA tag (Zinger et al., 2011) and subseafloor using a universal tag similar to V4 (Nunoura et al., 2016).

#### Comparison of Abundant Taxa

To examine the taxonomic composition of the samples, we chose the top 20 most abundant OTUs from each sample [similar to Inagaki et al. (2006), Agogué et al. (2011), and Petro et al. (2017)]. These taxa encompass all of the individual OTUs that are responsible for approximately 1% or more of the total sequences in each sample, which is a typical cutoff for considering OTUs to be abundant (Campbell et al., 2015; Inagaki et al., 2015; Tseng et al., 2015; Kirkpatrick et al., 2019). Jing et al. (2013) showed that using more than the 10 most abundant OTUs is sufficient to visualize changes in OTU community composition between samples. Our top OTUs account for 13–100% of the total sequences in each sample, with a total of 163 OTUs, and 170 OTUs for the V4 and V6 tag datasets, respectively.

**FIGURE 5:**
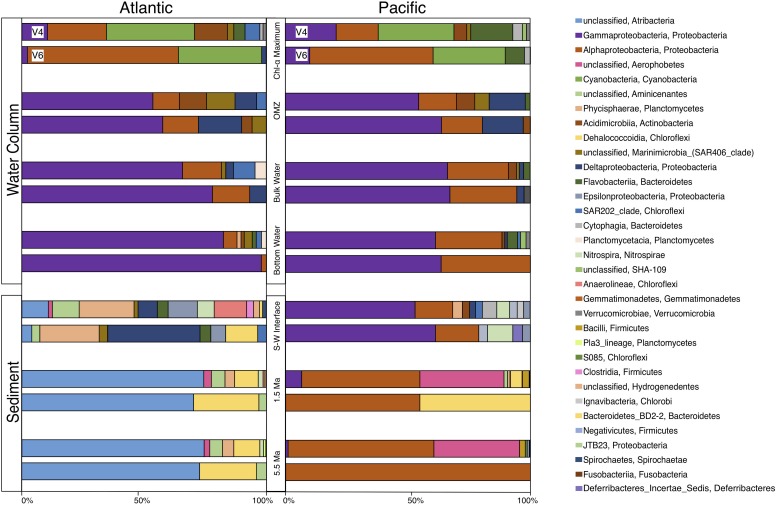
Class level community composition for the Atlantic and Pacific. At each depth horizon, the top bar displays community composition using the V4 tags and the bottom bar displays community composition using the V6 tags.

For a broad look at bacterial community composition, we separately grouped these 163 V4 tags and 170 V6 tags by taxonomic class (Figure 5). Although the total numbers of OTUs from each hypervariable region are similar (163 OTUs for V4 and 170 OTUs for V6), the number of class-level taxa returned from the V4 tags is almost double that of V6 (32 and 18 taxa, respectively). Most of this difference in richness is attributable to V4-based taxa that occur in low abundance and are from the Atlantic seafloor sediment sample, in particular. Except for this V4-based Atlantic community, the communities in all other samples are dominated by relatively few classes of bacteria and show a high degree of similarity between the V4 and V6 hypervariable region.

At both the Atlantic and Pacific sites, the water-column communities are broadly similar; they are dominated by *Cyanobacteria* and *Alphaproteobacteria* near the surface, sunlit water, and mostly *Gammaproteobacteria* in deeper water, similar to other 16S rRNA studies of water-column composition (DeLong et al., 2006; Brown et al., 2009; Jing et al., 2013; Tseng et al., 2015; Medina-Silva et al., 2018). In contrast, community composition in the sediment is quite different between the Atlantic and Pacific. Samples of older sediment in the Atlantic (dated approximately 1.5 and 5.5 Ma) are dominated by *Dehalococcoidia* and an unclassified *Atribacteria*, similar to other deep-sediment community composition studies (Inagaki et al., 2006, 2015; Blazejak and Schippers, 2010; Breuker et al., 2013; Teske et al., 2014; Brandt and House, 2016; Nunoura et al., 2016; Labonté et al., 2017; Petro et al., 2017; Orsi, 2018). In contrast, the communities in Pacific samples of the same age are primarily dominated by *Alphaproteobacteria*, an unclassified *Aerophobetes*, and some *Dehalococcoidia*. Inter-basin differences between the seafloor sediment samples are even more striking. The community of the Pacific seafloor sample is dominated by *Gammaproteobacteria* and *Alphaproteobacteria*, bearing a strong resemblance to the community composition in the overlying water column, as well as seafloor sediment from the South Atlantic (Schauer et al., 2010; Mills et al., 2012; Orsi et al., 2013; Bienhold et al., 2016; Jones et al., 2016) with additional, relatively rare taxa that give this sample its high taxonomic richness. As discussed above, the community in the Atlantic seafloor sample is quite diverse and other than one or two relatively abundant taxa, V4-based and V6-based community compositions are not in strong agreement. The V4-based Atlantic seafloor community contains a fairly even distribution of 14 taxa slightly dominated by *Phycisphaerae*, *Anaerolineae*, *Epsilonproteobacteria*, an unclassified *Aminicenantes*, and an unclassified *Atribacteria*. The V6-based community is not quite as evenly spread over eight taxa and dominated by *Deltaproteobacteria* and *Phycisphaerae*.

**FIGURE 6:**
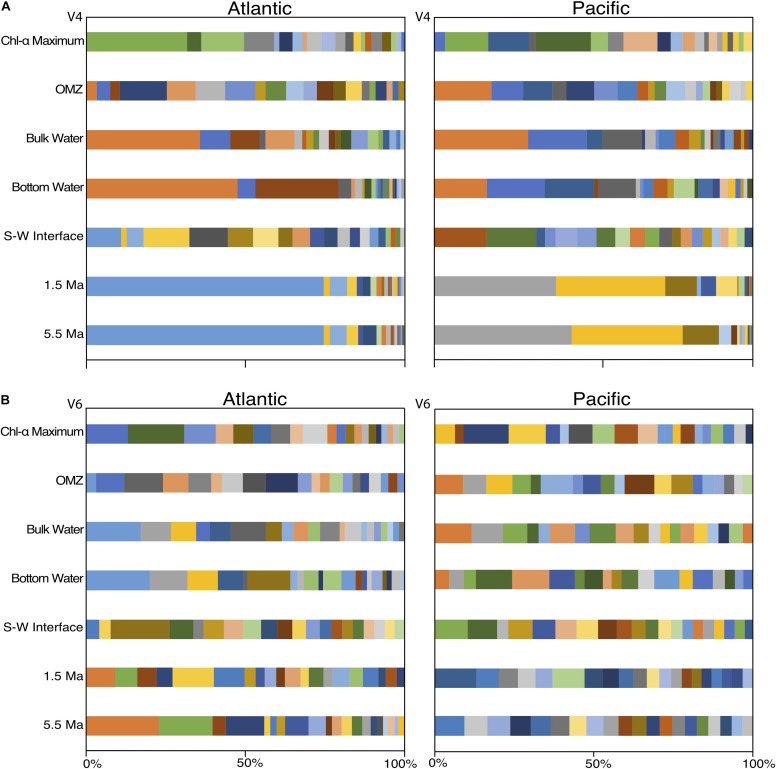
OTU level (97% similarity) community composition of the top 20 OTUs from the V4 hypervariable region **(A)** and the top 20 OTUs from the V6 hypervariable region **(B)**. Colors correspond to identical OTUs between the Atlantic and Pacific within hypervariable region, but not across regions.

To examine community composition at a finer taxonomic level, we plotted the vertical distributions of the 163 abundant V4-based OTUs and the 170 V6-based OTUs (Figure 6). The 163 V4-based OTUs exhibit a similar depth pattern of community composition in both the Atlantic and Pacific (as seen with the V4 class-level analysis), with between one and four dominant OTUs in each sample (Figure 6A). The water-column communities in both the Atlantic and Pacific are dominated by three V4-based OTUs of the genera *Halomonas*, *Idiomarina*, and *Erythrobacter* in the three mid-water samples, and two OTUs associated with photosynthetic metabolism of the genus *Prochlorococcus* in the upper, sunlit region, consistent with previous studies of the water column (DeLong et al., 2006; Brown et al., 2009; Campbell et al., 2015; Tseng et al., 2015). In the sediment samples, the Atlantic and Pacific communities are distinct. In the Atlantic, the subseafloor sediment (1.5 and 5.5 Ma) is dominated by one V4-based OTU associated with an unclassified genus of *Atribacteria*, whereas subseafloor sediment of the same age in the Pacific is dominated by two OTUs associated with the genus *Methylobacterium* and an unclassified genus of *Aerophobetes*. Consistent with the *E*_H_ values, the near-seafloor sedimentary communities exhibit very little bias toward any single V4-based OTU.

In contrast to the 163 V4-based OTUs (Figure 6A), and in contrast to the class-level V6-based analysis (Figure 5), the 170 V6-based OTUs do not exhibit clean depth-related patterns of taxonomic dominance (Figure 6B). Instead, many V6-based OTUs constituted a few percent of each sample, consistent with the calculated *E*_H_ values (Figure 3).

### Sampling Bias

Because the top 20 OTUs in each sample incorporate a greater percentage of total sequence reads for the V4-based analysis than for the V6-based analysis, it is possible that some of these disparities between the V4-based and V6-based results can be attributed to under-sampling of the V6-based communities by restricting the comparison to the top 20 OTUs in each sample. In order to test this possibility, we analyzed the top 200 V6 OTUs and compared them to the top 20 V4 OTUs in each of the three Atlantic sediment samples. We chose the three Atlantic sediment samples because they exhibit a consistent pattern in V4-based community composition, and might reasonably be expected to exhibit a similarly consistent pattern with V6 tags if under-sampling was the cause of the differences. We chose the top 200 V6 OTUs because it provides similar percentage coverage to the top 20 V4 tags for the same samples.

At the class level, this analysis led the number of *Phycisphaerae* reads in the V6 dataset to more closely match the number in the V4 dataset. However, it also added 15 classes to the V6 community not found in the V4 community, and it still produced very different compositions for the V4 and V6 seafloor communities (Supplementary Figure S1). The class-level V4-based and V6-based communities of the 1.5 and 5.5 Ma sediment samples in the Atlantic resemble each other more closely than do the class-level V4- and V6-based seafloor communities; this similarity of the subseafloor V4- and V6-based communities is mainly due to the dominance of *Dehalococcoidia* and unclassified *Atribacteria*, which were also similar with only the top 20 OTUs in each sample.

Despite the higher percentage of OTU coverage provided by the 200 most abundant V6 tags in each sediment sample, the top 200 V6-based OTUs still do not exhibit a clear pattern of taxonomic dominance (Supplementary Figure S2). This result agrees with the evenness metrics calculated for the V6 dataset, in which all the values are very high (*E*_H_ > 0.69 for all samples) and all OTUs, not just the 20 or 200 most abundant, are taken into consideration. This result suggests that the apparent differences in V4-based versus V6-based communities are not due to an under-sampling of the V6 dataset.

### Sub-Sampled Datasets

To compare the patterns of taxonomic richness and community composition from our deep sequencing run to those that would result from a shallow sequencing run, we randomly pulled 10,000 sequences from each sample and performed the same clustering and analysis as with the full dataset. While the absolute numbers of 97% similar OTUs were proportionally reduced for each sample, the patterns of relative OTU abundance remained the same as in the full dataset across both hypervariable regions and sample sites. Community composition was also not strongly affected, with the community composition pattern of the top 20 OTUs nearly identical to those for the full dataset. This result is consistent with previous studies that found no significant difference in diversity analysis between shallow and deep sequencing results (Kuczynski et al., 2010; Caporaso et al., 2012).

## Discussion

### Taxonomic Richness Patterns

All four of our vertical profiles of OTU richness (2 sites × 2 hypervariable regions) are similar in nature (Figure 1), indicating that the fundamental pattern in each of them broadly represents the relative richness of bacterial OTUs in the open ocean and marine sediment. In each case, water-column bacterial richness peaks in the OMZ and sedimentary richness peaks at the seafloor, in agreement with previous studies of the water column (Signori et al., 2014; Walsh et al., 2016b) and the sediment (Walsh et al., 2016b; Petro et al., 2017). For both hypervariable regions at both sites, taxonomic richness declines with sediment age, also in agreement with previous studies (Walsh et al., 2016a; Kirkpatrick et al., 2019). However, richness declines much more strongly in the Pacific V4 dataset than in the Atlantic V4 dataset, or either V6 dataset. This result suggests that sedimentary properties (e.g., geographic location, sediment composition, and sediment age) affect measures of taxonomic richness differently using different hypervariable regions. It also shows that in certain geographic locations, sediment up to 5.5-Ma in age may contain a large fraction of the taxonomic richness in the overlying water column.

### Taxonomic Richness Depends on Hypervariable Region

Although the V4 and V6 datasets are generally similar in their vertical profiles of taxonomic richness, the large difference between V4-based OTU numbers and V6-based OTU numbers at each sampling horizon illustrates that choice of one hypervariable region over another can significantly impact the number of clustered OTUs (Lebret et al., 2016). To further illustrate this inference, the V4-based richness values are strongly correlated to the V6-based richness values for each sample (*R*^2^ = 0.885) (Figure 2). However, the *y*-intercept of approximately 4500 on the V6 axis implies that V6 OTU richness is highly inflated. Previous studies have shown that V4-based taxonomic richness closely matches the OTU richness based on the entire 16S rRNA gene (Tremblay et al., 2015), whereas V6-based richness is consistently higher (Youssef et al., 2009). This dependence of OTU richness on hypervariable region complicates comparison studies that rely on different hypervariable regions. Such comparison is further complicated by a recent study which shows that, regardless of hypervariable region, clustering algorithms consistently group together sequences into single OTUs that would define separate OTUs using the entire 16S gene at the same similarity level (97%) (Mysara et al., 2017). Even when clustered at a more restrictive level (98 or 99%), the returned number of OTUs for different hypervariable regions is between 30 and 87% (this includes differences between hypervariable regions) of the OTU number for the entire 16S gene (Mysara et al., 2017). Their percentage of “overmerged” OTUs also varies between different bacterial families, and OTUs within each family are either more conserved, or less conserved, and depended on which hypervariable tag is analyzed (Mysara et al., 2017). Over- and under-merging species into OTUs depending on family and hypervariable region complicates discussion of absolute richness.

### Community Evenness

*E*_H_ values indicate that where the V4 tags define a community skewed toward one or two OTUs, these dominant V4-based OTUs may be broken down into several strains using the V6 tags. This result is particularly noticeable in the subseafloor sediment samples (from approximately 1.5 and 5.5 Ma), in which many V6-based OTUs merge into only a few V4-based OTUs. Additionally, the V6-based communities exhibit much greater evenness than the V4-based communities. Although 16S V6 clustering produces much higher numbers of 97% similar OTUs than V4 clustering or whole 16S clustering, V6 tag sequences still measure real differences in diversity (Liu et al., 2008). Consequently, a V6-based analysis will identify higher subseafloor diversity (and higher potential diversification) than a V4-based analysis (e.g., Starnawski et al., 2017).

### Community Composition

The PCoA clearly shows that community composition using 97% similar OTUs is similar for each environment in the water column and the sediment, regardless of the hypervariable region (V4 or V6) (Figure 4). This result indicates that even at the 97% similar OTU level, patterns of similarity or dissimilarity of community compositions obtained using a single hypervariable region are robust. The PCoA results also strengthen the finding that while V6 tag sequences return a higher number of 97% similar OTUs, the much higher evenness values of the V6-based community in the subseafloor sediment samples (Figure 3) are due to the much higher diversity of genetically similar reads in the V6 dataset relative to the V4 dataset, rather than hypervariable-region bias.

The V4-based communities and V6-based communities are broadly similar to each other at the class level (Figure 5). Regardless of the total number of 97% similar OTUs, each sample is dominated by the same class (or classes) of bacteria in both the V4-based and V6-based analyses. This similarity indicates that apparent community composition analysis of the most abundant taxa in a sample is not strongly affected by the hypervariable region chosen (Lebret et al., 2016). In our data, there are two distinct exceptions to this general pattern. First, in both the Atlantic and Pacific Chl-a maximum samples, the V4-based community is dominated by relatively similar numbers of *Gammaproteobacteria*, *Alphaproteobacteria*, and *Cyanobacteria*. However, the V6-based community is mostly dominated by *Alphaproteobacteria*, which is similar to other studies using only the V6 tag (Agogué et al., 2011; Zinger et al., 2011). Second, in the subseafloor sediment of both the Atlantic and Pacific, the V6-based community contains a high number of *Chloroflexi*, consistent with other subseafloor studies (Inagaki et al., 2006; Petro et al., 2017), whereas the V4-based community contains very few *Chloroflexi* reads, but includes a large percentage of *Firmicutes* that are completely absent in the V6-based community.

Despite the similarity of the V4 and V6 PCoA results and the visibly-similar patterns of class-level community compositions using the V4 and V6 datasets, strip charts of V4-based community compositions differ greatly from strip charts of V6-based community compositions at the level of 97% similar OTUs (Figure 6). The V4-based OTU communities exhibit a vertical pattern of composition similar to that at the class level, with dominance of each water-column or sediment sample by one or two OTUs in both the Atlantic and Pacific. The V4-based samples tend to be more visibly similar between separate samples taken from the same site and the same general environment (e.g., deep water or subseafloor sediment). For the V6-based communities, this visual similarity is obscured by high taxonomic richness and evenness. For example, the V4-based OTU community of the 1.5 Ma Atlantic subseafloor generally resembles the V4-based 5.5 Ma Atlantic community (Figure 6A), but the V6-based communities from the same samples appear to resemble each other much less closely (Figure 6B). In short, the community relatedness indicated by the PCoAs of the 97% similar OTUs is not readily visible in strip charts of V6-based community compositions. This result may be due to the much higher diversity of genetically similar reads in the V6 dataset relative to the V4 dataset.

This point can be further illustrated by examining a single, V4-based OTU in the oldest Atlantic sediment sample (5.5 Ma), identified as an unclassified *Atribacterium*. At the 97% similarity level, it is identical to the same V4-based OTU in the other two Atlantic sediment samples (seafloor and 1.5 Ma). However, examination of the same sample using the V6 tag reveals this V4-based *Atribacterium* OTU to contain 14 distinguishable V6-based OTUs, 13 of which were observed only in the two subseafloor sediment samples, and 7 of which were observed only in either the 1.5 sample or the 5.5 Ma sample. These sample-to-sample differences in presence or absence of V6 OTUs may be due to (i) under-sampling of the populations resident in the samples, (ii) variation in the V6 hypervariable region of 16S rRNA in the founding seafloor populations over the past 5.5 Myr, or (iii) diversification within the subseafloor community over the past 5.5 Myr which is detectible in the V6 region but not the V4 region. Identification of 6 of the 13 V6-based OTUs in both the 1.5-Ma sample and the 5.5-Ma sample indicates that the greater diversity in the V6 dataset is largely real, and not an artifact of sequencing error.

## Conclusion

Our comparison of paired V4 and V6 16S tags in natural samples of seawater and marine sediment confirms that estimates of bacterial OTU diversity and evenness depend on the 16S tag used. However, both tags yield similar patterns of taxonomic richness and evenness in the seawater and sediment at both the Atlantic site and Pacific site. And, when directly comparing the genetic distances of one sample from another (PCoA), the grouping of OTUs based on sample location is the same regardless of the tag used. Based on taxonomic assignment at the class level, community composition is broadly similar for both tags. However, at the 97% similar OTU level, the V4 and V6 tags yield different community compositions. This is perhaps because many marine bacteria are unclassified due to lack of a reference database. For both tags, water-column community composition is similar across geographic locations, but sediment community composition differs substantially from the Atlantic site to the Pacific site. Finally, deep sequencing provides no differences in patterns of relative diversity or in community composition of the most abundant taxa.

## Data Availability

The datasets generated for this study can be found in NCBI PRJNA423041.

## Author Contributions

ZK designed and executed the study with significant input from SD and JK, conducted all laboratory analyses and bioinformatics, with the exception of amplicon sequencing, and wrote the manuscript with significant input from SD. SD was the principal investigator. All authors provided editorial comments on the manuscript.

## Conflict of Interest Statement

The authors declare that the research was conducted in the absence of any commercial or financial relationships that could be construed as a potential conflict of interest.

## Footnotes

1 https://vamps.mbl.edu/resources/primers.php

## Supplementary Material

FIGURE S1 OTU level (97% similarity) community composition of the top 20 V4 OTUs compared to the top 200 V6 OTUs.

FIGURE S2 OTU level (97% similarity) community composition analysis of the top 200 OTUs based on V6 tag sequences for the Atlantic sediment.
